# Dedicated computer-aided detection software for automated 3D breast ultrasound; an efficient tool for the radiologist in supplemental screening of women with dense breasts

**DOI:** 10.1007/s00330-017-5280-3

**Published:** 2018-02-07

**Authors:** Jan C. M. van Zelst, Tao Tan, Paola Clauser, Angels Domingo, Monique D. Dorrius, Daniel Drieling, Michael Golatta, Francisca Gras, Mathijn de Jong, Ruud Pijnappel, Matthieu J. C. M. Rutten, Nico Karssemeijer, Ritse M. Mann

**Affiliations:** 1 Department of Radiology and Nuclear Medicine, Radboud University Medical Centre Nijmegen (NL), Geert Grooteplein 10, 6525 GA Nijmegen, The Netherlands; 2 Department of Biomedical Imaging and Image Guided Therapy, Division of Molecular and Gender Imaging, Medical University of Vienna/Vienna General Hospital (A), Vienna, Austria; 3 Department of Radiology, Centre Diagnosi per la Imatge Tarragona (E), Tarragona, Spain; 4 Center for Medical Imaging and Department of Radiology, University Medical Centre Groningen (NL), Groningen, Netherlands; 5 MeVis Medical Solutions, Bremen (DE), Bremen, Germany; 6 Department of Gynaecology and Obstetrics, Universitäts-Frauenklinik Heidelberg (D), Heidelberg, Germany; 7 Department of Radiology, Jeroen Bosch Hospital, s-Hertogenbosch (NL), s-Hertogenbosch, Netherlands; 8 Department of Radiology, University Medical Centre Utrecht (NL), Utrecht, Netherlands

**Keywords:** Ultrasonography, Breast neoplasms, Diagnosis, Computer-assisted, Mammography, Early detection of cancer

## Abstract

**Objectives:**

To determine the effect of computer-aided-detection (CAD) software for automated breast ultrasound (ABUS) on reading time (RT) and performance in screening for breast cancer.

**Material and methods:**

Unilateral ABUS examinations of 120 women with dense breasts were randomly selected from a multi-institutional archive of cases including 30 malignant (20/30 mammography-occult), 30 benign, and 60 normal cases with histopathological verification or ≥ 2 years of negative follow-up. Eight radiologists read once with (CAD-ABUS) and once without CAD (ABUS) with > 8 weeks between reading sessions. Readers provided a BI-RADS score and a level of suspiciousness (0-100). RT, sensitivity, specificity, PPV and area under the curve (AUC) were compared.

**Results:**

Average RT was significantly shorter using CAD-ABUS (133.4 s/case, 95% CI 129.2-137.6) compared with ABUS (158.3 s/case, 95% CI 153.0-163.3) (*p* < 0.001). Sensitivity was 0.84 for CAD-ABUS (95% CI 0.79-0.89) and ABUS (95% CI 0.78-0.88) (*p* = 0.90). Three out of eight readers showed significantly higher specificity using CAD. Pooled specificity (0.71, 95% CI 0.68-0.75 vs. 0.67, 95% CI 0.64-0.70, *p* = 0.08) and PPV (0.50, 95% CI 0.45-0.55 vs. 0.44, 95% CI 0.39-0.49, *p* = 0.07) were higher in CAD-ABUS vs. ABUS, respectively, albeit not significantly. Pooled AUC for CAD-ABUS was comparable with ABUS (0.82 vs. 0.83, *p* = 0.53, respectively).

**Conclusion:**

CAD software for ABUS may decrease the time needed to screen for breast cancer without compromising the screening performance of radiologists.

**Key Points:**

• *ABUS with CAD software may speed up reading time without compromising radiologists’ accuracy.*

*• CAD software for ABUS might prevent non-detection of malignant breast lesions by radiologists.*

*• Radiologists reading ABUS with CAD software might improve their specificity without losing sensitivity.*

## Introduction

In mammographic screening the sensitivity in women with extremely dense breasts is only 61% [1]. A four times higher interval cancer rate is reported for these women compared with women with fatty breasts [1]. Supplemental ultrasound (US) is an effective imaging method to detect mammography-negative early stage invasive breast cancer in women with heterogeneously and extremely dense breasts [2–4], thus reducing the frequency of symptomatic interval carcinomas [5]. This is crucial, because detection of breast cancer at an early stage substantially improves prognosis, even when using modern therapy regimes [6]. This explains the rationale and ratification of the breast density inform laws in many states in the USA [7, 8] and the introduction of supplemental whole-breast ultrasound (WBUS) screening in Austria [9].

Performing supplemental WBUS with handheld devices has limitations. It is relatively time consuming and difficult to compare to prior examinations. Furthermore, handheld WBUS screening is operator dependent and should therefore be performed by trained sonographists, which consequently requires substantial resources [10]. Automated 3D breast US (ABUS) devices have been developed to improve the reproducibility of WBUS and decrease the need for highly trained sonographers. An ABUS examination consists of a set of large 3D volumes for each breast acquired with a wide automatically driven linear array transducer. The number of volumes depends on the size of the breast and in large breasts up to five volumes per breast are acquired. There is mounting evidence that, similar to handheld ultrasound, ABUS devices also lead to the detection of mammography-negative invasive breast cancers [11–15].

A downside of supplemental ultrasound screening is the detection of mammographically occult benign lesions that warrant histological verification [11, 13, 16], thus decreasing the specificity of screening. ABUS devices do allow storage of full breast ultrasound volumes, which enables the radiologist to compare examinations with relevant priors, which is expected to improve specificity in follow-up examinations.

Due to the large number of images in the scan, reading a full ABUS examination can be lengthy and cancers may easily be overlooked [12]. Computer-aided detection (CAD) software for ABUS has been developed to aid radiologists in the interpretation of ABUS studies [17]. CAD software should reduce the reading time of supplemental ABUS and may have the potential to improve the screening performance of radiologists. To investigate the effectiveness of this approach, we investigated the effect of commercially available CAD software for ABUS on the reading time and screening performance of breast radiologists.

## Materials and methods

The need for informed consent for this study was waived by the institutional review board (IRB).

### ABUS acquisitions

ABUS examinations were performed with ACUSON S2000 Automated Breast Volume Scanner systems (Siemens, Erlangen, Germany). This ABUS system acquires 3D B-mode ultrasound volumes over an area of 154 mm × 156 mm using a mechanically driven linear array transducer (14L5). Adequate depth and focus can be obtained using predefined settings for different breast cup sizes. All ABUS examinations were performed by technicians. To ensure coverage of the entire breast two to five overlapping acquisitions were performed at predefined locations. The number of acquisitions depends on the size of the breasts and the possibility to compress the breasts. Per acquisition 318 slices of 0.5 mm thickness are obtained. A dedicated ABUS workstation reconstructs the transverse slices into a 3D volume that can be read in a multiplanar hanging, also showing sagittal and coronal reconstructions.

### Data and gold standard

Cases were selected from a large multi-institutional imaging archive that consisted of 2158 ABUS examinations in 1086 women acquired between August 2010 and February 2015 from screening programmes for women at average, intermediate, and high risk and symptomatic women. For each woman a full-field digital mammography (FFDM) examination was also available.

To select only cases with high breast density, breast density was determined using an automated volumetric software package (Volpara Density, Matakina Ltd. Wellington, New Zealand) on 1657 available unprocessed FFDM images. For 501 examinations, where unprocessed FFDM images were not available, breast density was visually assessed according to the BIRADS lexicon. Examinations of 115 women with a history of breast surgery were excluded; 1187 unilateral examinations of breasts in 715 women were scored as Volpara Density Grade 3 and 4 or BIRADS density categories C or D. We categorised these dense cases as “normal” (*n* = 919), “benign” (*n* = 140), or “malignant” (*n* = 128) based on radiology and pathology reports from histopathological examinations. “Normal” and non-biopsied “benign” cases were only considered if at least 2 years of negative follow-up was available. Subsequently, from these women with dense breasts, we included all cases with a mammography-negative malignant lesion (*n* = 20), ten randomly selected malignant cases that were positive on both mammography and ABUS, 30 biopsied benign cases and 60 “normal” cases in the study data set. The study data set thus consisted of 120 unilateral ABUS evaluations, yielding a total of 375 ABUS volumes.The selected cases were anonymised and stripped from information such as age, study date, and imaging institute. All lesions were annotated by a breast imaging researcher with > 3 years of experience with ABUS based on pathology and radiology reports. These annotations served as the ground truth for observer and CAD software detection performance.

### CAD software and reading workstation

**Fig. 1:**
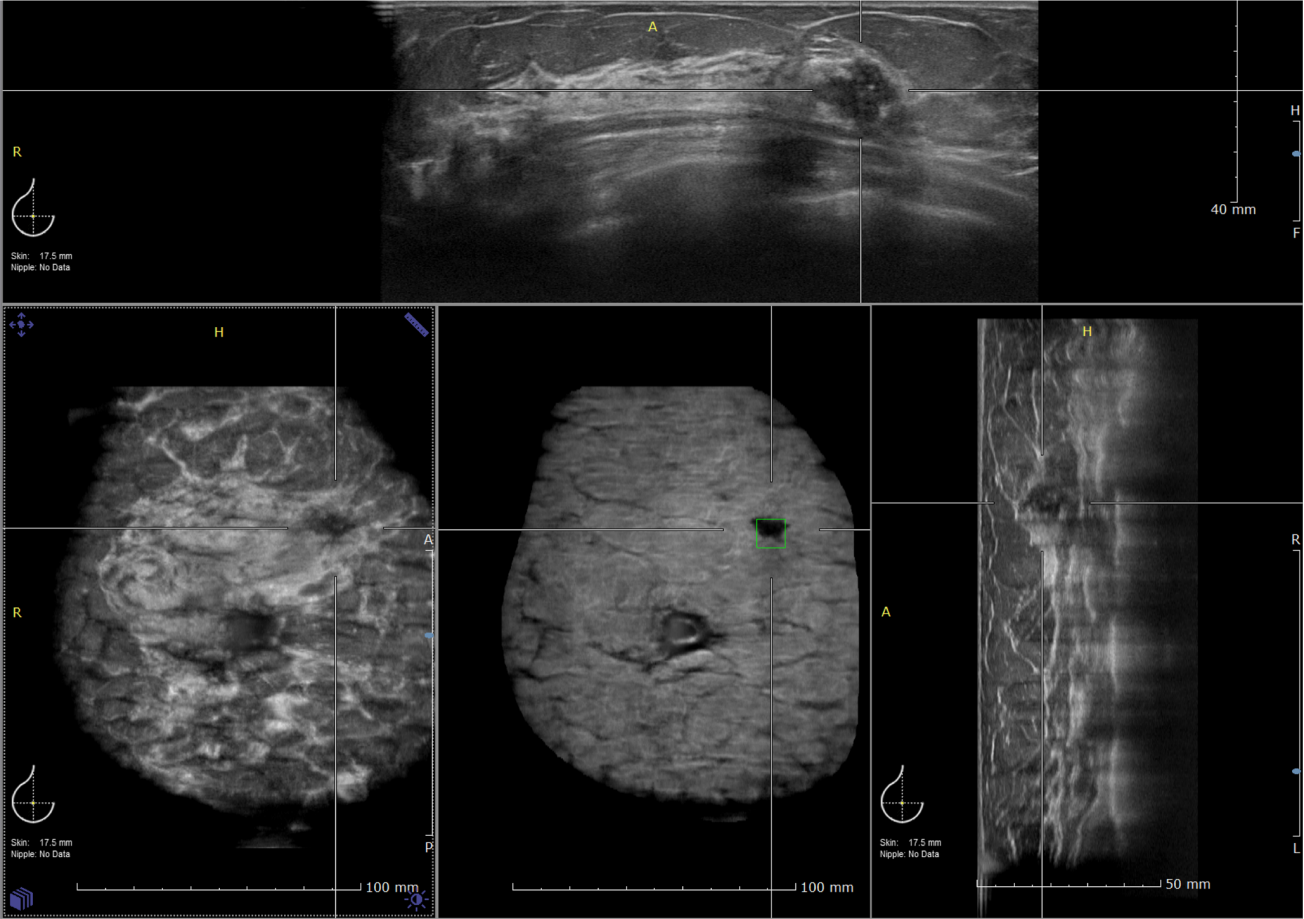
CAD-based minimum intensity projection (MinIP) integrated in a multiplanar hanging protocol for ABUS that shows the conventional ABUS planes. The top plane shows the transverse acquisitions, the lower left plane the coronal reconstructions, and the lower right plane the sagittal reconstruction. The MinIP (bottom row in the middle) is a 2D image where lower intensity regions in the 3D ABUS volume are enhanced as dark spots. By clicking on the dark spot, the 3D multiplanar hanging automatically snaps to the corresponding 3D location. The CAD marks (coloured square) are displayed on the MinIP

A prototype workstation was designed and developed specifically for the task of high-throughput ABUS screening in this observer study (MeVis Medical Solutions, Bremen, Germany). In this prototype, each user action was logged with time stamps that were subsequently used to estimate the time spent per case. Commercially developed CAD software (QVCAD, Qview Medical Inc., Los Altos, CA) was integrated into this workstation. This CAD software is designed to detect suspicious region candidates in an ABUS volume and mark them with so-called CAD marks (Fig. 1). In addition, QVCAD software provides an “intelligent” minimum intensity projection (MinIP) of the breast tissue in a 3D ABUS volume that can be used for rapid navigation through ABUS scans and enhances possible suspicious regions. The number of CAD marks displayed can be adjusted by setting the average number of false-positive CAD marks per ABUS volume. In this study, we chose the default setting of one false-positive CAD mark per ABUS volume.

### Readers

Seven breast radiologists and one gynaecologist specialised in breast imaging were invited to participate in this study. By inviting readers from different institutes and countries we aimed to increase the applicability of our results to breast imaging practices in different countries, realising that different readers might have slightly varying standards and customs. In some countries, also other clinicians are involved in interpreting breast-imaging examinations. Therefore, we also invited a non-radiologist (gynaecologist) who specialises in breast imaging with approximately 10 years of experience in breast ultrasound and mammography and 8 years of experience with ABUS. Experience with breast imaging for reader one to reader eight was 7, 10, 4, 8, 8, 20, 4, and 20 years and specifically with ABUS was 5, 8, 0, 5, 5, 5, 0, and 0 years, respectively.

### Study design

All eight readers evaluated all cases twice in two separate reading sessions in an independent crossover multi-reader-multi-case (MRMC) study. In each session half of the ABUS cases were read conventionally and half of the cases were read using a CAD-based workflow designed for this study. We counterbalanced the reading modes and changed the case order by randomisation for each reader per reading session. The reading sessions were at least 8 weeks apart (average 11.0 weeks, range 8.3-13.1) to further minimise any effect of memory bias.

Standard ABUS reading was performed in a multiplanar hanging without CAD software. CAD-based reading was performed according to specific instructions of a two-step reading protocol. The first step was to evaluate all CAD marks and dark spots on the MinIP in a case. Subsequently, readers were instructed to scan the coronal reconstruction of each ABUS view in a hanging protocol where coronal reconstructions of all ABUS views of a breast are simultaneously shown.

The readers performed a training session of 20 cases to become familiar with the workstation, reading protocol, and CAD software. Readers were given a rough estimate (10-30%) of the prevalence of cancer in the study data set because the criteria for a recall may vary between radiologists who, as in our study, work at different institutes and in different countries [18] and may depend on the prevalence of cancers they expect.

In both CAD-based and conventional reading the readers were instructed to mark and rate lesions by placing a finding marker and subsequently determine a BI-RADS assessment score. Because a quasi-continuous linear scale is required to perform receiver-operating characteristic (ROC) analysis, readers were also asked to provide a level of suspiciousness (LOS) score on a scale from 0-100. Note that LOS is not a probability of malignancy as described in the BI-RADS atlas. Instead, readers were recommended to use anchor points referring to the BI-RADS scores with LOS values of 21, 41, 61, and 81 corresponding to the BI-RADS 1/2, 2/3, 3/4, and 4/5 transitions.

### Statistical analysis

We determined the sensitivity, specificity, and positive predictive value (PPV) in both reading modes based on BI-RADS scores and compared these parameters per reader using paired McNemar’s and chi-square tests with bootstrapping (1000 samples) to determine the 95% confidence intervals (CI) for individual readers and generalised estimation equation (GEE) for pooled data to correct for repeated measurements. An examination was considered positive if a BI-RADS 3 score (and its anchor point equivalent of 41 on the LOS scale) or higher was given. Furthermore, we determined the area under the curve (AUC) and 95 % CI using an alternative free-response receiver-operating characteristics (AFROC) [19, 20]. For these analyses, when multiple findings were present in a case, the finding with the highest rating was used. Ratings in malignant cases where the marker was placed outside of the annotated lesion margin were not included in the analysis and regarded as a false negative (missed cancer). By doing so, readers are not rewarded for a recall based on a false-positive finding accidentally occurring in a malignant case. We compared the AUCs for both reading modes for each reader individually and also pooled over all readers (random readers, random cases). Reading time was compared for each reader individually by using Student’s *t*-test with 1000 bootstraps to determine the 95% CI and GEE for pooled data. Only the readings recorded within the 95th percentile were included in the analysis to correct for inactivity of the reader during the reading sessions.

The ROC analyses were performed using MRMC software (JAFROC, version 4.2.1). The GEE was performed using the ‘geese’ function in the ‘geepack’ package in R (v. 3.2.3, R Foundation for Statistical Computing, Vienna, Austria). All other analyses were performed with SPSS statistics 20.0 (IBM Statistics, Armonk, NY).

## Results

### Patient characteristics

Table 1 summarises the patient characteristics in women with breast cancer and Table 2 summarises patient characteristics of women with a “normal” or “benign” ABUS examination.

**Table 1:** Characteristics of the malignant cases in the data set

Malignant cases	Mean age (SD)	N Symptomatic:screening	N FFDM neg:pos	Mean lesion size in mm (SD)	Lymph node metastasis	HR+ HER2-	HR+ HER2+	HR- HER2+	HR- HER-	Unknown receptor status	†Grade I	†Grade II	†Grade III	Grade unknown
Total (*n* = 30)	49.8 (12.1)	17:13	20:10	16.0 (8.8)	8	16	2	3	4	4	4	14	10	2
Invasive ductal carcinoma (*n* = 22)	48 (11.1)	15:7	14:8	16.9 (9.9)	6	11	2	3	3	3	2	10	8	2
Invasive lobular carcinoma (*n* = 3)	73.5 (4.9)	1 : 2	2 : 1	14.7 (5.5)	1	3	0	0	0	0	0	3	0	0
Invasive metaplastic carcinoma (*n* = 2)	47.0 (14.1)	1 : 1	1 : 1	16.5 (2.1)	0	0	0	0	1	0	0	0	2	0
Invasive tubular carcinoma (*n* = 1)	52	0 : 1	1 : 0	7	0	1	0	0	0	0	1	0	0	0
Invasive intracystic papillary carcinoma (*n* = 1)	45	0 : 1	1 : 0	12	1	1	0	0	0	0	1	0	0	0
Non-invasive intracystic papillary carcinoma (*n* = 1)	49	0 : 1	1 : 0	14	0	0	0	0	0	1	0	1	0	0

^†^Nottingham histological grade (modified Bloom-Richardson-Elston)

FFDM Full-field digital mammography

HR Hormone receptor status (oestrogen and progesterone receptors)

HER2 Human epidermal growth factor receptor 2 status

**Table 2:** Characteristics of women with an ABUS examination labelled as ‘benign’ and ‘normal’

	Mean age (SD)	*N* symptomatic:screening	Mean size (SD)
Normal cases (*n* = 60)	42.0 (9.5)	4:56	N/A
Benign cases total (*n* = 30)	44.9 (9.1)	15:15	12.4 (5.1)
Fibroadenoma (*n* = 12)	42.9 (5.3)	7:5	12.4 (5.7)
Fibrosis/adenosis (*n* = 5)	43.6 (6.3)	1:4	10.2 (4.1)
Cystic lesions (*n* = 5)	46.6 (8.8)	3:2	14.8 (7.8)
Other benign breast tissue (*n* = 5)	54.6 (13.0)	3:2	12.2 (1.9)
Papilloma (*n* = 2)	38.5 (9.2)	1:1	14.0 (2.8)
Complex sclerosing lesion (*n* = 1)	30.0	0:1	8.0 (0.0)

SD Standard deviation

### Screening performance

**Fig. 2:**
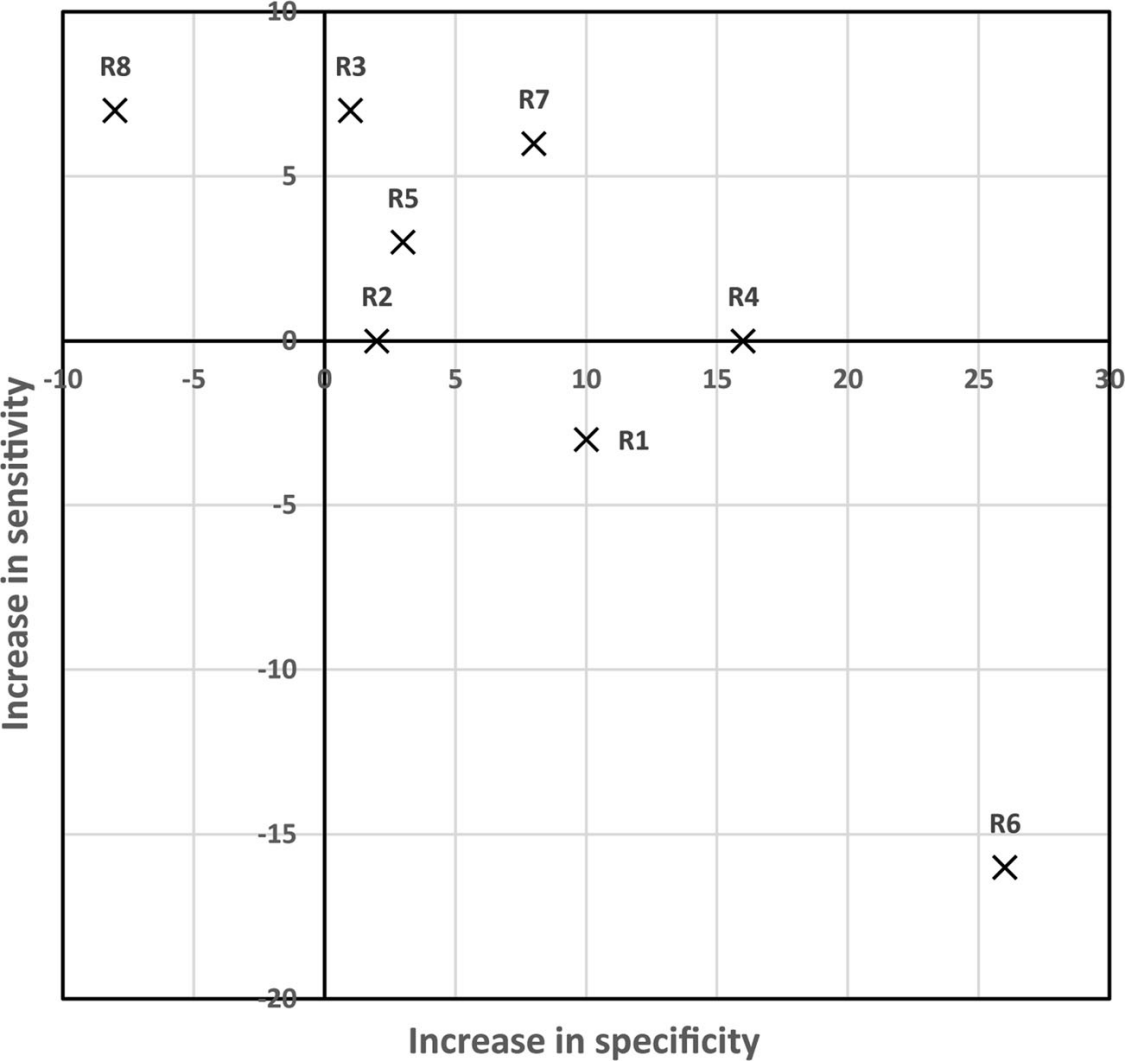
Increment in sensitivity and specificity per reader after subtracting the sensitivity of the specificity of the conventional ABUS reading session from the CAD-based workflow reading session. Ideally all readers perform within the upper right quadrant

Figure 2 and Table 3 summarise the screening performance per reader. On average, the sensitivity of unaided conventional ABUS reading (84%, 95% CI 78-88) was similar to the sensitivity in the CAD-based ABUS reading protocol (84%, 95% CI 79-89) (*p* = 0.90). Nevertheless, half of the readers detected more cancers with CAD, while only two readers detected fewer cancers using the CAD-based reading protocol. In the CAD-based readings 6 out of 8 readers placed markers on a total of 11 lesions that were actually malignant, but still classified them as benign (BI-RADS 2). In the unaided ABUS reading this happened only in four readers and a total of five malignant lesions. Hence CAD helped in the detection of additional cancers but could not always induce an adequate classification by the readers.

**Table 3:** Individual performance per reader for the conventional ABUS reading and the CAD-based workflow reading

Reader (years of ABUS experience)		Sensitivity	95% CI (up, low)		*p*- value	Specificity	95% CI (up, low)		*p* value	PPV	95% CI (up, low)		*p* value	AUC	95% CI (up, low)		*p* value
1 (5)	*ABUS*	0.80	0.67	0.93		0.79	0.71	0.88		0.56	0.42	0.70		0.77	0.64	0.91	
	*CAD*	0.77	0.60	0.90	1.00	0.89	0.82	0.96	0.03	0.70	0.55	0.85	0.22	0.83	0.71	0.94	0.34
2 (8)	*ABUS*	0.83	0.67	0.97		0.69	0.60	0.79		0.47	0.34	0.60		0.79	0.66	0.92	
	*CAD*	0.83	0.7	0.97	1.00	0.71	0.62	0.80	0.82	0.49	0.35	0.63	0.85	0.8	0.67	0.93	0.93
3 (0)	*ABUS*	0.73	0.57	0.87		0.73	0.64	0.82		0.48	0.35	0.61		0.73	0.60	0.87	
	*CAD*	0.80	0.63	0.93	0.63	0.74	0.66	0.83	1.00	0.51	0.36	0.64	0.76	0.78	0.67	0.90	0.27
4 (5)	*ABUS*	0.80	0.63	0.90		0.64	0.54	0.74		0.43	0.30	0.55		0.85	0.75	0.95	
	*CAD*	0.80	0.63	0.90	1.00	0.80	0.71	0.88	0.001	0.57	0.43	0.71	0.16	0.87	0.78	0.96	0.51
5 (5)	*ABUS*	0.87	0.73	0.97		0.68	0.58	0.77		0.47	0.35	0.60		0.88	0.79	0.98	
	*CAD*	0.90	0.80	1.00	1.00	0.71	0.61	0.80	0.70	0.51	0.38	0.64	0.70	0.87	0.77	0.97	0.79
6 (5)	*ABUS*	0.93	0.83	1.00		0.42	0.32	0.52		0.35	0.25	0.45		0.87	0.79	0.96	
	*CAD*	0.77	0.60	0.90	0.06	0.68	0.58	0.78	< 0.001	0.44	0.31	0.58	0.29	0.81	0.71	0.91	0.21
7 (0)	*ABUS*	0.87	0.73	0.97		0.74	0.66	0.83		0.53	0.39	0.67		0.88	0.78	0.96	
	*CAD*	0.93	0.83	1.00	0.5	0.82	0.74	0.90	0.19	0.64	0.48	0.77	0.30	0.92	0.85	0.99	0.21
8 (0)	*ABUS*	0.83	0.70	0.97		0.51	0.41	0.61		0.36	0.25	0.48		0.81	0.70	0.92	
	*CAD*	0.90	0.80	1.00	0.5	0.43	0.33	0.53	0.35	0.35	0.24	0.46	0.84	0.81	0.71	0.91	0.96
Pooled	*ABUS*	0.84	0.78	0.88		0.67	0.64	0.70		0.44	0.39	0.49		0.82	0.73	0.92	
	*CAD*	0.84	079	0.89	0.90	0.71	0.68	0.75	0.08	0.50	0.45	0.55	0.07	0.83	0.75	0.92	0.53

Sensitivity, specificity, and PPV are based on the BI-RADS assessment per case. The AUC is based on a BI-RADS-based linear rating scale from 0-100

ABUS Automated *b*reast *u*ltrasound reading

CAD Computer*-a*ided *d*etection*-*based workflow reading

PPV Positive predictive value (for all recommendations other than routine screening follow*-*up)

AUC Area under the curve

95% CI 95% confidence interval

**Fig. 3:**
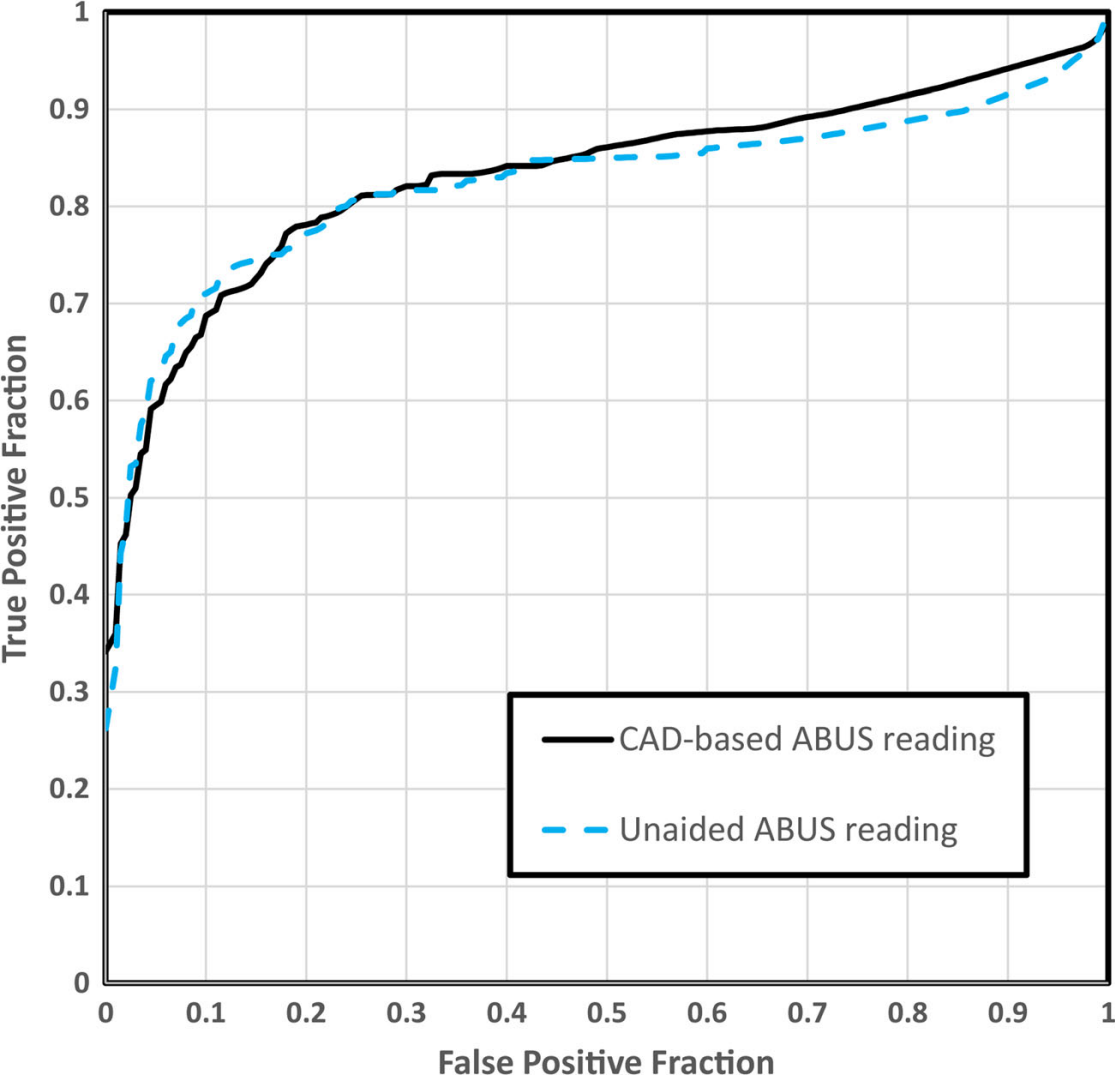
Alternative free-response receiver-operating characteristic curves for conventional ABUS reading (striped intervals) and computer-aided detection based workflow reading (straight line). No statistical difference is observed between the areas under the curves

The average specificity for conventional ABUS reading was 67% (95% CI 64-70) and this increased to 71% (95% CI 68-75) in the CAD-based reading strategy, although this did not reach statistical significance (*p* = 0.08). The PPV was on average 13.6% higher for the CAD-based ABUS reading (50.0%, 95% CI 45-55) compared to the conventional ABUS reading (44.0%, 95% CI 39-49) (also not significant, *p* = 0.07). Overall, seven out of eight readers had higher specificity and PPV with CAD than without. Specificity was significantly higher in three out of eight readers (readers 1, 4, and 6; Table 3). Nevertheless, the AUCs did not statistically differ between the conventional ABUS reading and the CAD-based workflow (0.82, 95% CI 0.73-0.92 and 0.83, 95% CI 0.75-0.92, respectively) (*p* = 0.53) (Fig. 3).

### Reading time

**Fig. 4:**
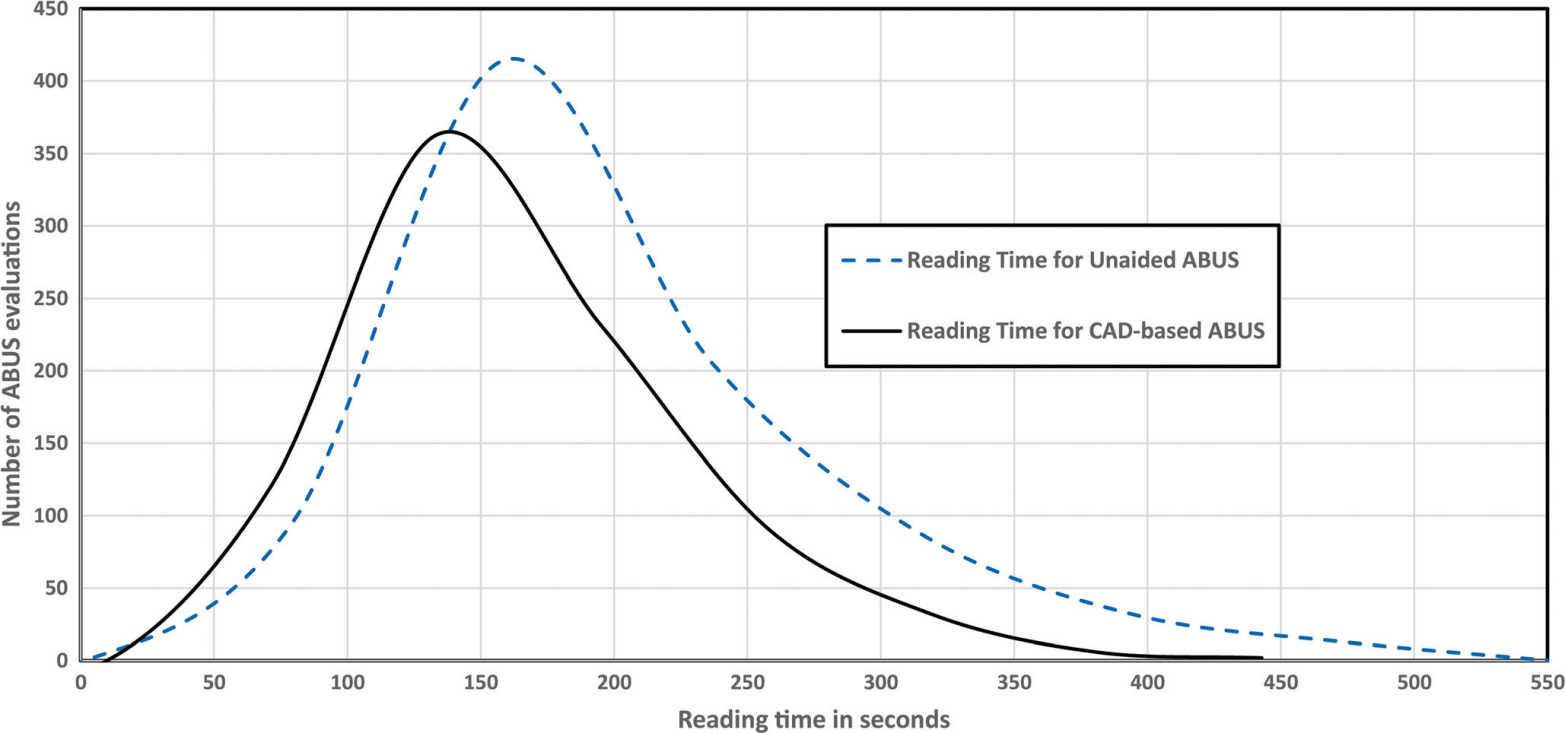
Histograms for reading time needed to read all cases in a conventional ABUS protocol (striped interval) and for reading in a CAD-based workflow protocol (straight)

Table 4 summarises the reading time for each individual reader. On average, reading unilateral ABUS examinations using CAD software decreases the overall reading time by 24.9 s/case (SE 3.43; *p* < 0.001) (Fig. 4), which is a reduction of 15.7%. All readers were faster using CAD software (range, 3.1%-26.3%). In six out of eight readers, the CAD-based workflow was significantly faster.

**Table 4:** Average reading time per reader for both conventional ABUS reading and reading the CAD-based reading workflow

Reader (years experience ABUS)	Average reading time ABUS (s)	95% CI (low, high)		Average reading time CAD-ABUS (s)	95% CI (low, high)		Percentage decrease	*p* value
1 (5)	171.2	156.5	186.5	166.0	150.4	181.0	3.1	0.56
2 (8)	145.4	132.4	159.1	136.1	124.5	149.6	6.5	0.24
3 (0)	146.7	132.6	162.2	123.4	113.0	134.3	15.9	< 0.001
4 (5)	175.2	158.7	190.8	140.8	130.2	150.1	19.7	0.001
5 (5)	101.2	95.7	108.4	91.2	84.7	97.7	9.9	0.008
6 (5)	138.6	127.1	151.1	110	100.1	119.4	20.6	0.001
7 (0)	217.2	197.9	236.2	160.1	148.0	172.3	26.3	0.001
8 (0)	173.3	173.3	185.2	140.9	132.3	150.0	18.7	0.001
Pooled								
Average	158.3	153.0	163.6	133.4	129.2	137.6	15.7	< 0.001
Normal	151.0	143.6	158.4	125.7	120.0	131.4	16.8	< 0.001
Benign	163.0	152.6	173.3	134.8	126.4	143.1	17.3	< 0.001
Malignant	169.3	158.8	180.0	148.8	140.2	157.5	12.1	0.003

All readers were faster with CAD software. Six of eight readers were significantly faster* ABUS Automated breast ultrasound*

*CAD Computer-aided detection software*

The average reading time for malignant cases decreased by 12.1% (20.5 s/case, SE 6.97, *p* = 0.003), for benign cases by 17.3% (28.2 s/case, SE 6.77, *p* ≤ 0.001), and for normal cases by 16.8% (25.3 s/case, SE 4.76) (*p* ≤ 0.001).

## Discussion

Our study shows that CAD software for ABUS can help radiologists to evaluate ABUS examinations more efficiently. Radiologists who screen for breast cancer may use CAD software to evaluate batches of ABUS examinations 15.7% faster, without decreasing their performance in terms of cancer detection. Interestingly, the higher specificity and PPV of the CAD-based reading mode suggest that the use of CAD software for ABUS may help radiologists avoid unnecessary recalls of healthy women, albeit this did not reach statistical significance. Our results might facilitate further implementation of ABUS. Supplemental ABUS in women with mammographically dense breasts helps radiologists detect early stage cancers that are occult on mammography [11–13]. Supplemental US screening reduces the interval cancer rate in women with dense breasts [2, 21], which in general is associated with improved outcome [6]. Unfortunately, 31% of cancers in supplemental US screening are found to be already visible on a prior screening US examination and could still have been detected earlier [22]. Reasons for non-detection in WBUS screening are usually misinterpretation and oversight errors. In our study, oversight errors in malignant cases were more often observed in conventional ABUS reading than in the CAD-based reading. In fact, half of the readers detected and correctly classified more cancers in the CAD-based readings than in conventional ABUS reading. Nevertheless, of the missed cancers several were still marked by six readers in the CAD-based reading, but wrongly classified as benign. Therefore, it appears that the CAD software has the potential to prevent oversight errors in ABUS but might require further development to also aid in characterising lesions. Also the very limited experience all readers had with the CAD system might have partly contributed to the misclassification of malignant lesions.

Supplemental ABUS has been shown to increase the recall rate in breast cancer screening programmes [11, 13]. The implementation of an intelligent MinIP into the reading environment therefore also aims at improvement of specificity. The MinIP uses the greyscale contrast in B-mode ultrasound between lesions and healthy tissue to summarise the 3D volume in a 2D image; hence normal tissue appears lighter than cancers that show up as dark spots on the MinIP. Moreover the CAD software also enhances the more suspicious regions by lowering the intensity of the lesion on the MinIP and strengthening the coronal retraction sign, which is highly suggestive of breast cancer in ABUS [23]. Consequently, the MinIP points out relevant lesions and reduces the suspiciousness of irrelevant regions in ABUS volumes. Our study indicates that using this CAD software might indeed decrease unnecessary recalls in ABUS by improving the specificity and PPV of radiologists. Although the overall results were not significant, a positive effect was still seen in seven out of eight readers. Whether ABUS CAD software in actual supplemental screening truly helps to decrease the recall rate and improve radiologist’s specificity still needs to be investigated prospectively.

In a previous pilot study, we investigated the effect of CAD software for ABUS on the screening performance of readers when screening for breast cancer [24]. Our previous study showed that concurrent reading CAD software may improve the accuracy of radiologists for evaluation of single ABUS volumes. In the current study, the CAD software was implemented into a specific CAD-based screening workflow to boost the reading speed during batch reading of whole-breast ABUS examinations. The purpose of this study was therefore to investigate the effect of CAD software on the efficiency rather than on the accuracy. In addition, this study was performed using whole-breast examinations only from women with heterogeneously dense or extremely dense breasts, thus creating a data set that is representative for supplementary screening with ABUS in dense breasts. The mean reading time of a unilateral ABUS examination with an average of three volumes per breast without CAD software in our study was 158.3 s, which is in line with previously reported 3-9 min for a bilateral WBUS examination [11, 16, 25]. However, our study data set was enriched with cancers and suspicious benign cases, which likely increases the reading time per case. Our CAD-based reading workflow decreased the average reading time with 15.7% to 133.4 s per unilateral ABUS examination. The improvement in reading speed was higher in normal and benign cases than in malignant cases. We therefore expect that this gain in efficiency in a true screening setting could be higher than in our study.

Navigation of the ABUS examinations using the CAD-enhanced MinIP can be performed relatively quickly. But in our study the readers were instructed to evaluate all dark spots and CAD marks in the MinIP and subsequently also scan the coronal reconstructions of the ABUS volumes. As a consequence our instructions prolonged the reading time in the CAD-based reading sessions. Most breast radiologists are familiar with the concept of summarising relevant information of 3D breast imaging in a 2D image, as is common practice in tomosynthesis (synthetic mammogram) and in dynamic contrast-enhanced breast MRI [maximum intensity projections (MIP)]. Kuhl et al. reported that looking only at MIPs is a reliable and fast (3-30 s per case) approach to breast cancer screening with MRI [26]. The CAD-enhanced MinIP in our study could theoretically be used in a similar way, thus further reducing the reading time required per ABUS volume. However, future studies need to elucidate the effect this may have on the sensitivity of ABUS.

Our study has limitations. We did not show corresponding mammograms with the ABUS examinations although these modalities are complementary in most screenings regimes of women with dense breasts and this might positively or negatively affect the screening performance. Furthermore, we enriched the data set with benign and malignant lesions from both screening and diagnostic examinations to increase the power in this study. By doing so, our study data set does not represent clinical practice where the prevalence of benign and malignant lesions is lower. Finally, multiple readers had little experience with ABUS and all readers were inexperienced with the CAD software package that we implemented in our screening environment, which may have negatively affected the screening performance and reading time.

In conclusion, our study shows that the CAD software developed for ABUS has the potential to improve the efficiency of reading ABUS by significantly improving the reading speed without decreasing the screening performance. Further research is warranted in a prospective study to investigate the effect of CAD on breast cancer detection, screening recalls, and the interval cancer rate in screening programmes.

## Abbreviations

ABUS: Automated breast ultrasound
ABVS: Automated breast volume scanner
AFROC: Alternative free-response receiver-operator characteristics
ANOVA: Analysis Of variance
AUC: Area under the ROC curve
CAD: Computer-aided detection
FFDM: Full-field digital mammography
GEE: Generalised estimation equation
HER2: Human epidermal growth factor receptor 2 status
HR: Hormone receptor status
IRB: Institutional review board
LOS: Level Of suspiciousness
MinIP: Minimum intensity projection
MIP: Maximum intensity projection
MRI: Magnetic resonance imaging
MRMC: Multiple reader multiple case
PPV: Positive predictive value
ROC: Receiver-operator characteristics
RT: Reading time
US: Ultrasound
WBUS: Whole-breast ultrasound
